# Exploring the Relationship Between Smartphone GPS Patterns and Quality of Life in Patients With Advanced Cancer and Their Family Caregivers: Longitudinal Study

**DOI:** 10.2196/59161

**Published:** 2025-02-07

**Authors:** Kyungmi Lee, Andres Azuero, Sally Engler, Sidharth Kumar, Frank Puga, Alexi A Wright, Arif Kamal, Christine S Ritchie, George Demiris, Marie A Bakitas, J Nicholas Odom

**Affiliations:** 1 Frances Payne Bolton School of Nursing, Case Western Reserve University, Cleveland, OH, United States; 2 School of Nursing, University of Alabama at Birmingham, 1720 2nd Avenue South, Birmingham, AL, 35294, United States, 1 205-934-7597; 3 Department of Computer Science, University of Alabama at Birmingham, Birmingham, AL, United States; 4 Department of Medical Oncology, Harvard Medical School, Dana-Farber Cancer Institute, Boston, MA, United States; 5 Department of Medicine, Duke University, Durham, NC, United States; 6 Mongan Institute Center for Aging and Serious Illness, Division of Palliative Care and Geriatric Medicine, Massachusetts General Hospital and Harvard Medical School, Boston, MA, United States; 7 School of Nursing & Department of Biostatistics, Epidemiology and Informatics, Perelman School of Medicine, University of Pennsylvania, Philadelphia, PA, United States; 8 Division of Geriatrics, Gerontology, and Palliative Care, Department of Medicine, University of Alabama at Birmingham, Birmingham, AL, United States; 9 Center for Palliative and Supportive Care, University of Alabama at Birmingham, Birmingham, AL, United States

**Keywords:** cancer, digital phenotyping, global positioning system, quality of life, smartphone, mobile phone, family caregiver

## Abstract

**Background:**

Patients with advanced cancer and their family caregivers often experience poor quality of life (QOL). Self-report measures are commonly used to quantify QOL of family caregivers but may have limitations such as recall bias and social desirability bias. Variables derived from passively obtained smartphone GPS data are a novel approach to measuring QOL that may overcome these limitations and enable detection of early signs of mental and physical health (PH) deterioration.

**Objective:**

This study explored the feasibility of a digital phenotyping approach by assessing participant adherence and examining correlations between smartphone GPS data and QOL levels among family caregivers and patients with advanced cancer.

**Methods:**

This was a secondary analysis involving 7 family caregivers and 4 patients with advanced cancer that assessed correlations between GPS sensor data captured by a personally owned smartphone and QOL self-report measures over 12 weeks through linear correlation coefficients. QOL as measured by the Patient-Reported Outcomes Measurement Information System (PROMIS) Global Health 10 was collected at baseline, 6, and 12 weeks. Using a Beiwe smartphone app, GPS data were collected and processed into variables including total distance, time spent at home, transition time, and number of significant locations.

**Results:**

The study identified relevant temporal correlations between QOL and smartphone GPS data across specific time periods. For instance, in terms of PH, associations were observed with the total distance traveled (12 and 13 wk, with *r* ranging 0.37 to 0.38), time spent at home (−4 to −2 wk, with *r* ranging from −0.41 to −0.49), and transition time (−4 to −2 wk, with *r* ranging −0.38 to −0.47).

**Conclusions:**

This research offers insights into using passively obtained smartphone GPS data as a novel approach for assessing and monitoring QOL among family caregivers and patients with advanced cancer, presenting potential advantages over traditional self-report measures. The observed correlations underscore the potential of this method to detect early signs of deteriorating mental health and PH, providing opportunities for timely intervention and support.

## Introduction

Each year, an estimated 1.96 million individuals are expected to receive a cancer diagnosis in 2023 [1]. Projections indicate a near 25% increase in cancer diagnoses by 2032 when it is estimated that 22.5 million US individuals will have a history of cancer [2]. With cancer care transitioning increasingly to home settings, the impact of cancer will increasingly stretch beyond the individual diagnosed to those family and friend caregivers who provide support [3]. Consequently, family caregivers have become indispensable frontline clinicians for patients, especially when their cancer progresses to advanced stages [4]. Both patients and their family caregivers face an array of challenges, from logistical and practical challenges to managing treatments and symptoms to handling a range of strong emotions, including anxiety and depression [5]. Hence, it is not surprising that patients and their caregivers often experience diminished quality of life (QOL) [6-8], which cancer care clinicians aim to assess and optimize.

Traditionally, assessing QOL has primarily relied on patient-reported outcome measures (PROMs) using paper-and-pencil questionnaires, online surveys, or verbal self-report to a data collector reading from a list of items [9,10]. However, PROMs have several notable limitations [11,12]. They can be time consuming for both patients and clinicians and interrupt daily activities. Furthermore, they typically entail retrospective recall which can be subject to distorting biases [13,14] and may elicit socially desirable responses, such as over-reporting better health or greater well-being than actually experienced [15-17].

To address the limitations of a PROM approach to assessing patient and caregiver QOL states, digital phenotyping has emerged as a new and potentially innovative approach. Defined as the “moment-by-moment quantification of the individual-level human phenotype in situ using data from personal digital devices” [18], this method involves passive data collection from ubiquitously owned devices such as smartphones, now owned by approximately 85% of US individuals [19]. By using smartphone sensor data, digital phenotyping models changes in individuals’ behaviors, including mobility (eg, GPS data), sociability (eg, text message and telephone logs), and sleep patterns (eg, accelerometer data and screen activity logs) [10].

Physical activity has been shown to improve both the physical and mental well-being of patients with cancer and their caregivers [20,21]. Recent studies have demonstrated that engaging in regular physical activity reduces the risk of cancer recurrence and alleviates symptoms of anxiety and depression, which in turn positively influence QOL [22,23]. These benefits are particularly important for caregivers, who often experience high levels of stress, as physical activity can improve their mental health (MH) and overall well-being [24].

GPS data has been shown to correlate with measures assessing mood, anxiety, and depression [25]. Better mood, lower anxiety, and reduced depression were associated with spending more time at locations of friends and family members. Conversely, more time spent at health care facilities was linked to poorer mood, higher anxiety, and increased depression [25]. Furthermore, GPS data were examined in comparing recovery metrics between mastectomy and breast-conserving surgery in patients with breast cancer [26]. The results indicated that patients undergoing mastectomy spent more time at home in the initial weeks postsurgery, whereas no significant differences were observed after 12 weeks. Despite a growing body of research that highlights the relationship between individual GPS-derived mobility behaviors and MH and well-being [27,28], a digital phenotyping approach to QOL assessment has yet to be explored in an advanced cancer context.

This study aimed to explore the temporal relationship between smartphone GPS sensor features and participants’ physical health (PH) and MH over a 12-week period. We hypothesized that more consistent and stable GPS-derived mobility features would correlate with better patient and caregiver PROM reports of QOL.

## Methods

### Study Design

This was a secondary analysis of data collected from patient and caregiver participants of a larger, ongoing prospective longitudinal observational study [29]. This analysis used data collected over 12 weeks from individuals enrolled between September 2021 and August 2022 at an academic medical center in the southeastern United States as part of a larger research project investigating patterns in passively collected smartphone behavioral data, including GPS and accelerometer.

### Ethical Considerations

This study is a secondary analysis of deidentified data collected from a prior study, which was approved by the University of Alabama at Birmingham Institutional Review Board (IRB; 300008890). The IRB determined that this secondary analysis does not constitute Human Subjects Research and is exempt from further IRB review. Details regarding the informed consent process and participant compensation in the original study are described in the Procedures section. For this secondary analysis, no direct participant interaction occurred, and the dataset was fully deidentified before access. Therefore, no additional informed consent or participant compensation was required.

### Sample

We included 7 family caregivers and 4 patients with advanced cancer who were recruited as of August 4, 2022. Inclusion criteria for both caregivers and patients were (1) aged 18 years or older, (2) owned a personal smartphone (Android or iOS) and had the ability to download and run the study app, and (3) had proficiency in the English language (spoken and written). In addition, enrolled caregivers provided regular support to a patient with advanced cancer, while patients had to be diagnosed with metastatic, recurrent, or progressive stage III or IV cancer.

### Procedures

Once potential participants were identified, they received an invitation letter containing the study description and a timeline of study activities through mail. Subsequently, participants were contacted by telephone to confirm their interest. Verbal consent was obtained during these calls, and participants were provided with an informed consent document, baseline questionnaires, and a participant guide. After completing the baseline questionnaire, research coordinators assisted participants in downloading and installing the Beiwe app on their personally owned smartphone, which passively collected GPS data. Patient-reported questionnaires were completed through various modalities, including electronic completion through REDCap (Research Electronic Data Capture; Vanderbilt University), mailed paper-and-pencil surveys, or phone-based data collection. Both caregiver and patient participants received US $25 for phone usage and an additional US $25 for each completed questionnaire every 6 weeks.

### Beiwe Research Platform

For this research, we used the Beiwe Research Platform, which has a primary focus on developing statistical and computational tools to extract biomedical and clinical insights from smartphone data [30]. The Beiwe app, developed by Torous and colleagues [30], passively collects smartphone GPS data and securely synchronizes it over Wi-Fi to a cloud-based server managed by Beiwe on Amazon Web Services. The platform includes a study portal, applications for both Android and iOS, secure data storage on Amazon Web Services, and data analysis tools and code [30]. To prioritize privacy, passive data undergo encryption both during transit and storage, and indirect identifiers, such as telephone numbers and IP addresses, are hashed, a process that converts the original data into a fixed-size string of characters to enhance the security and privacy of the data [30]. Data linkage is exclusively tied to an 8-character Beiwe participant ID to ensure confidentiality [30].

### Data Collection

#### Demographics

Collected demographic information included age, gender, race or ethnicity, religion, marital status, household size, employment and education levels, relationship status, caregiving duration, and caregiving frequency.

#### QOL Data

Health-related QOL was assessed using the Patient-Reported Outcomes Measurement Information System (PROMIS) Global-10 questionnaire at baseline, 6 weeks, and 12 weeks. This questionnaire assesses respondents on their perception of overall health, pain, fatigue, social health, MH, and PH [9,31]. Two subscale scores are generated for PH (α=.86) and MH (α=.81), respectively [32]. Lower scores (MH <40, PH <42) indicated poor MH and PH [33], and are associated with higher risk of future health care use [34,35].

#### GPS Data

We established a data-quality dataset indicating the “valid” GPS hours for each participant per day, enabling the identification and exclusion of individuals with insufficient data during analyses [36]. Our threshold for valid GPS data was set at 15 hours per day, considering that participants might sleep for 9 hours during which GPS records may not be transmitted [37]. Missing location data resulting from issues such as phone battery depletion or power-off states were excluded cases. We determined the number of days with a minimum of 15 hours of recorded GPS data for each user in our dataset. Subsequently, days meeting this hourly cutoff were extrapolated to 24 hours. Additionally, for week-level analyses, participants with fewer than 3 valid days per week were excluded from our analysis [36].

### Data Analysis and Feature Extraction From GPS Data

#### Overview of Feature Extraction

For data analysis, we used Python (version 3.9.0; Python Software Foundation) in Jupyter Notebooks and Google Collaboratory to preprocess the GPS data and extract mobility variables, including total distance, time spent at home, transition time, and number of significant locations.

#### Total Distance

This variable measured the total distance covered by each participant using the Haversine distance formula, which calculates the angular distance between 2 points on the Earth’s surface [38].

#### Time Spent at Home

The home cluster for each participant was identified based on two heuristics: (1) it was among the top 3 most visited clusters, and (2) it was the cluster most visited between 12 AM and 6 AM [39]. The area of the participant’s home was assumed to be in a circular area with a 2000 square feet area from the estimated latitude and longitude of the house location. The time spent within this 2000 square feet area was calculated for each day.

#### Transition Time

Location data points were categorized as either stationary (eg, working in an office) or transitional (eg, walking on the street) based on movement speed [39]. The time derivative of each data point was used to calculate the movement speed, and a threshold speed of 1 meter per second (m/s) was applied to distinguish between transitional (speed>1 m/s) and stationary states (speed<1 m/s) [39]. The time spent at speeds greater than 1 m/s was calculated for each day.

#### Number of Significant Locations

This feature represents the total number of clusters found using a machine learning clustering algorithm [40]. The algorithm, called adaptive k-means, classified data points into specific groups based on their locations during stationary states [41]. The goal was to identify places where participants spent most of their time, such as homes, workplaces, and parks. The algorithm used the elbow method to determine the optimal number of clusters, which helps minimize the overall distances of data points to the centers of their respective clusters [42].The number of significant locations visited was then calculated for each day.

### Overview of Data Analysis

#### Statistical Methods Used

Data analysis was conducted using the R programming language (version 4.2.1, R Foundation), in RStudio (RStudio, PBC). Descriptive statistics were computed for variables of interest, and patterns of missing data were explored. PROMIS Global-10 mental and PH T-scores were used as measures of QOL. The association between GPS location features and patient-reported QOL was assessed using linear correlation coefficients. Temporal association between QOL assessments and GPS data were calculated weekly, using repeated-measures correlations with 1000 cluster-bootstrap resamples [43] to estimate 95% CIs. Cluster bootstrapping [44] was applied to handle multiple observations per participant.

#### Temporal Correlations

**Figure 1.**
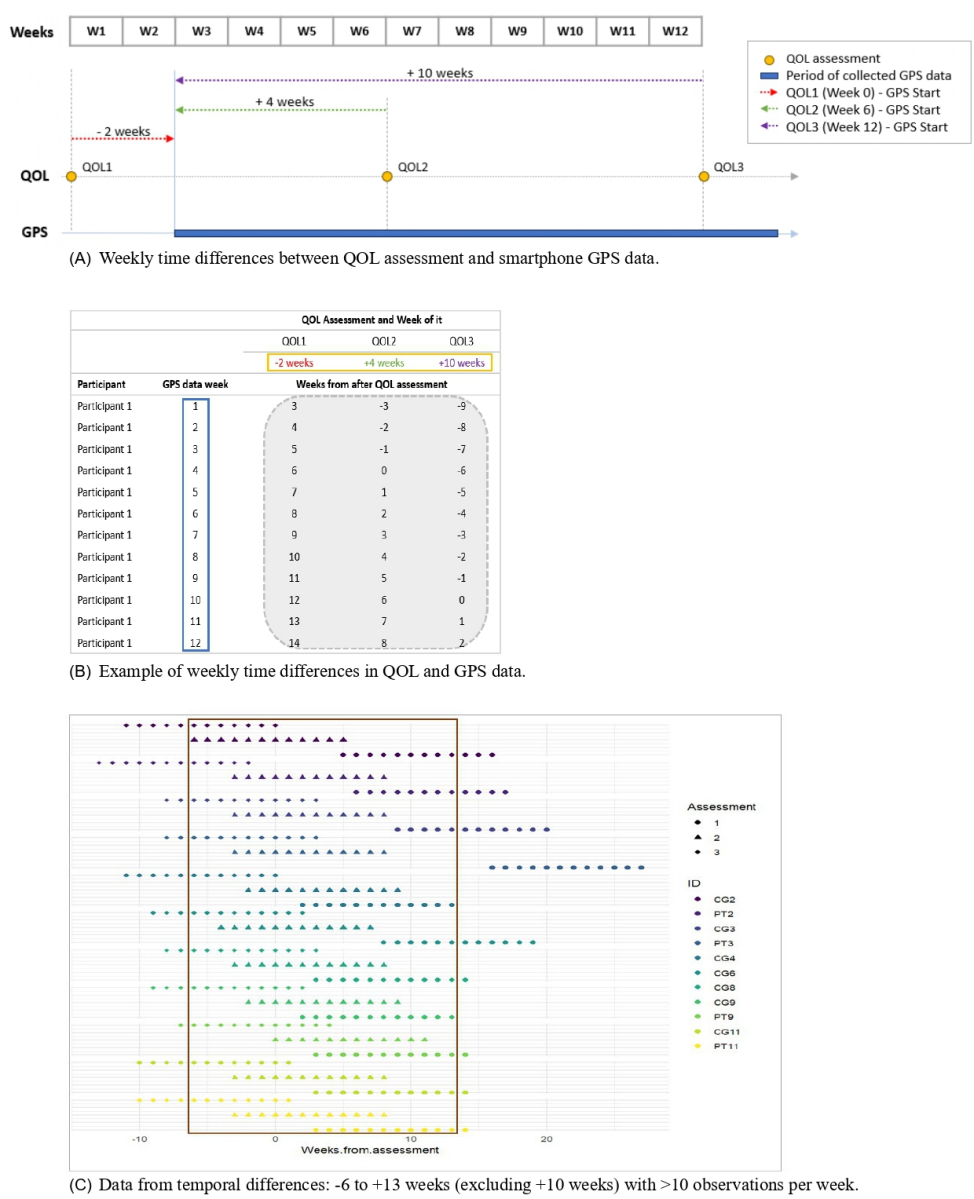
Time differences between patient-reported QOL assessments and daily smartphone GPS data were calculated on a weekly basis, as QOL measurements did not align with the smartphone data collection weeks of 0, 6, and 12. (A) Weekly time differences between QOL assessment and smartphone GPS data. (B) Example of weekly time differences in QOL and GPS data. (C) Data from temporal differences: –6 to +13 weeks (excluding +10 weeks) with >10 observations per week. QOL: quality of life.

As the QOL measurements did not necessarily align with weeks 0, 6, and 12 of smartphone data, we calculated the time difference between each patient-reported QOL assessment and daily smartphone GPS data on a weekly basis. Relative to the week of QOL assessment collection, time differences between smartphone data and QOL assessments ranged from −15 weeks to +14 weeks. The number of observations available for estimating a correlation at each temporal difference was computed. We considered data for temporal differences with more than 10 available observations per week, resulting in analysis for −6 to +13 weeks, excluding +10 weeks (Figure 1).

## Results

### Demographics of Participants

The study included 11 participants (7 caregivers and 4 patients). Caregivers had a mean age of 47.7 (SD 15.9) years, while patients had a mean age of 55 (SD 3.7) years. Most participants identified as White (10/11, 90%) and non-Hispanic or non-Latino (11/11, 100%). Most participants (9/11, 82%) identified as Protestant, were married (10/11, 90%), employed (5/11, 45%), and had a high school diploma or General Educational Development or higher (10/11, 90%). Among caregivers, 71% (5/7) cared for a spouse, and their average caregiving duration was 557 (SD 96) days. Most caregivers (6/7, 86%) lived with the patients. Regarding insurance, 75% (3/4) of patients had private or commercial medical insurance, and 25% (1/4) had military insurance. Furthermore, 75% (3/4) of patients resided in rural areas (Table 1).

**Table 1.** Demographics of participants.

Variables	Caregivers (n=7)	Patients (n=4)	All (N=11)
Age (years), mean (SD)	47.71 (15.86)	55 (3.65)	50 (13)
Gender, n (%)			
Male	3 (43)	2 (50)	5 (46)
Female	4 (57)	2 (50)	6 (55)
Race, n (%)			
Black or African American	1 (14)	0 (0)	1 (9)
White	6 (86)	4 (100)	10 (90)
Hispanic or Latino, n (%)			
Yes	0 (0)	0 (0)	0 (0)
No	7 (100)	4 (100)	11 (100)
Religion, n (%)			
Protestant	6 (86)	3 (75)	9 (82)
Catholic	0 (0)	1 (25)	1 (9)
None	1 (14)	0 (0)	1 (9)
Marital status, n (%)			
Never married	1 (14)	0 (0)	1 (9)
Married	6 (86)	4 (100)	10 (90)
Employment status, n (%)			
Full time	4 (57)	0 (0)	4 (36)
Part time	0 (0)	1 (25)	1 (9)
Unemployed due to disability or illness	1 (14)	1 (25)	2 (18)
Homemaker	1 (14)	1 (25)	2 (18)
Other	1 (14)	1 (25)	2 (18)
Education, n (%)			
≤8th grade	1 (14)	0 (0)	1 (9)
Some high school	0 (0)	0 (0)	0 (0)
High school graduate or General Educational Development	2 (29)	2 (50)	4 (36)
Some college or technical school	2 (29)	0 (0)	2 (18)
College graduate (bachelor’s degree)	2 (29)	2 (50)	4 (36)
Relationship with patient, n (%)			
Spouse	5 (71)	—^a^	—
Parent	1 (14)	—	—
Other	1 (14)	—	—
Lives with patient, n (%)			
Yes	6 (86)	—	—
No	1 (14)	—	—
Length of caregiving (day), mean (SD)	556.57 (96)	—	—
The number of days of care per week, n (%)			
Every day of the week (7 days)	3 (43)	—	—
2 to 3 days a week	2 (29)	—	—
1 day a week or less	2 (29)	—	—
Hours of care per day, n (%)			
5 to 6 hours a day	1 (14)	—	—
3 to 4 hours a day	1 (14)	—	—
1 to 2 hours a day	5 (71)	—	—
Medical insurance, n (%)			
Private or commercial	—	3 (75)	—
Military program	—	1 (25)	—
Residence, n (%)			
Urban	—	0 (0)	—
Suburban	—	1 (25)	—
Rural	—	3 (75)	—

a Not applicable.

### QOL Assessment Results

#### PH Findings

At baseline, the mean PH score across all participants was 47.1 (SD 6.7). Caregivers had a slightly lower mean PH score (mean 45.4, SD 4.6) compared to patients (mean 50.1, SD 9.4). Around 18% (2/11) of participants reported baseline PH T-scores below 42, including 1 caregiver and 1 patient. By the 6-week mark, the average PH score had increased to 49 (SD 9.6), with caregivers reporting a mean score of 50.2 (SD 10.3) and patients a mean score of 47.1 (SD 9.2). Similarly, around 18% (2/11) of participants recorded 6-week PH T-scores below 42, including 1 caregiver and 1 patient. At the 12-week assessment, the overall mean PH score slightly decreased to 46.6 (SD 6.8), with caregivers and patients reporting mean scores of 46.8 (SD 6.1) and 46.3 (SD 9.1), respectively. This time, around 27% (3/11) of participants, including 1 caregiver and 2 patients, had 12-week PH T-scores below 42 (see Multimedia Appendix 1 for participant-reported QOL results).

#### MH Findings

At baseline, the mean MH score across all participants was 50.7 (SD 8.6). Caregivers had a slightly lower mean MH score (mean 47.9, SD 7.7) compared to patients (mean 55.5, SD 8.8). One caregiver (9%) reported a baseline MH T-score below 40. At the 6-week evaluation, the mean MH score across all participants slightly increased to 51.5 (SD 7.7), with caregivers and patients reporting mean scores of 51.1 (SD 9) and 52.2 (SD 6), respectively. By the 12-week mark, the overall mean MH score further improved to 52.8 (SD 7.2), with caregivers at a mean score of 52.2 (SD 7.3) and patients at 53.9 (SD 8.1). One caregiver (9%) reported a 12-week MH T-score below 40 (Multimedia Appendix 1).

### Daily Smartphone GPS Data Weekly Averages

Over the 12-week period, the total distance variable ranged from a minimum of 102.2 (SD 66.5) km to a maximum of 186.7 (SD 153.2) km. The time spent at home variable ranged from a minimum of 437.2 (SD 388.5) minutes to a maximum of 573.3 (SD 271.1) minutes. The transition time variable ranged from a minimum of 800.4 (SD 225.3) minutes to a maximum of 903.4 (SD 109.4) minutes. The number of significant locations variable ranged from a minimum of 4.2 (SD 1.4) to a maximum of 5.1 (SD 1.6; see Multimedia Appendix 2 for details on daily smartphone GPS location features averaged by week).

### Correlation Between PH and Daily Smartphone GPS Data Over a 12-Week Period

**Figure 2.**
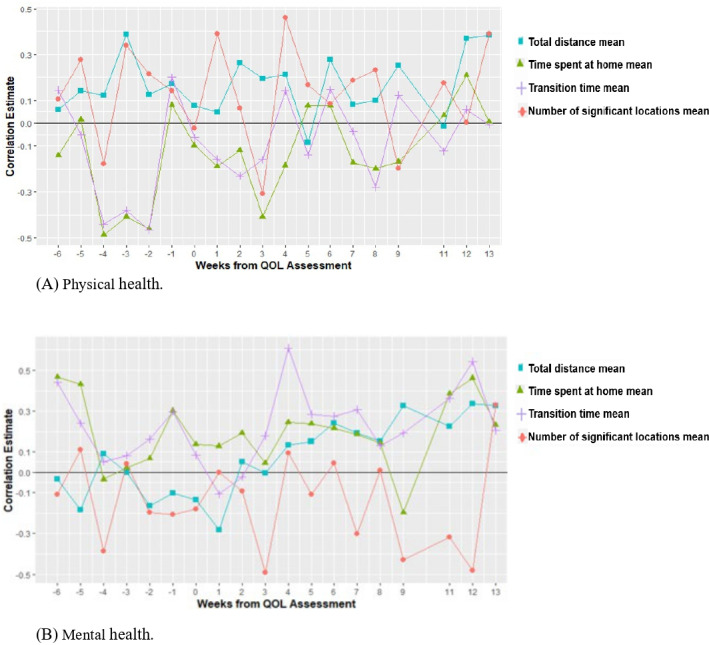
Correlation between physical and mental health and daily smartphone GPS features, including total distance, time spent at home, transition time, and number of significant locations averaged by week and using 95% cluster-bootstrap CIs over a 12-week period. (A) Physical health. (B) Mental health. QOL: quality of life.

Table 2 illustrates the temporal correlation between PH and weekly GPS location features. Significant correlations, with a value of +0.3 or higher, were observed between total distance and PH at 3 weeks before and during weeks 12 and 13 after the QOL assessment. Positive correlations at 3 weeks before and during weeks 12 and 13 after the QOL assessment imply that increased distances correlate with higher PH. Correlations of −0.3 or higher were found between time spent at home and PH at 2, 3, and 4 weeks before the QOL assessment, as well as at the 3 weeks after the assessment. These negative correlations suggest a potential association between increased time spent at home and lower PH. Similarly, correlations of −0.3 or higher were found between transition time and PH at 2, 3, and 4 weeks before the QOL assessment. These negative correlations also suggest a potential association between increased transition time and lower PH. Moreover, correlations of ±0.3 or higher between the number of significant locations and PH were significant at 3 weeks before and at weeks 1, 3, and 4 after the QOL assessment. Negative correlations at week 3 after the QOL assessment suggest that as the number of places visited increases, PH tends to decline, while positive correlations at week 3 before and at weeks 1 and 4 after the QOL assessment indicate a potential link to higher PH (Figure 2).

**Table 2.** Correlation between physical health and daily smartphone GPS features, including total distance, time spent at home, transition time, and number of significant locations, averaged by week and using 95% cluster-bootstrap CIs over a 12-week period.

From QOL^a^, weeks^b^	Observations, n	Participants, n	Total distance mean, *r*^c^ (95% CI)	Time spent at home mean, *r* (95% CI)	Transition time mean, *r* (95% CI)	Number of significant locations mean, *r* (95% CI)
−6	11	10	0.06 (−0.42 to 0.74)	−0.14 (−0.52 to 0.41)	0.14 (−0.31 to 0.63)	0.11 (−0.34 to 0.58)
−5	11	10	0.14 (−0.47 to 0.54)	0.02 (−0.26 to 0.38)	−0.05 (−0.56 to 0.35)	0.28 (−0.14 to 0.76)
−4	12	10	0.12 (−0.37 to 0.61)	−0.49 (−0.71 to −0.05)	−0.44 (−0.84 to 0.06)	−0.18 (−0.48 to 0.35)
−3	16	11	0.39 (0.01 to 0.65)	−0.41 (−0.69 to −0.02)	−0.38 (−0.74 to 0.04)	0.34 (−0.06 to 0.63)
−2	16	10	0.12 (−0.44 to 0.63)	−0.46 (−0.80 to 0.02)	−0.47 (−0.80 to 0.01)	0.21 (−0.23 to 0.66)
−1	19	11	0.17 (−0.40 to 0.76)	0.08 (−0.26 to 0.61)	0.20 (−0.15 to 0.57)	0.14 (−0.20 to 0.57)
0	19	11	0.08 (−0.54 to 0.53)	−0.10 (−0.47 to 0.24)	−0.06 (−0.41 to 0.18)	−0.02 (−0.28 to 0.29)
1	18	11	0.05 (−0.44 to 0.62)	−0.19 (−0.45 to 0.10)	−0.16 (−0.45 to 0.13)	0.39 (0.11 to 0.66)
2	19	11	0.26 (−0.54 to 0.80)	−0.12 (−0.47 to 0.33)	−0.23 (−0.46 to −0.03)	0.07 (−0.26 to 0.47)
3	20	11	0.20 (−0.21 to 0.60)	−0.41 (−0.65 to −0.20)	−0.16 (−0.66 to 0.31)	−0.31 (−0.55 to 0.43)
4	16	11	0.21 (−0.16 to 0.71)	−0.18 (−0.50 to 0.17)	0.14 (−0.31 to 0.63)	0.46 (0.13 to 0.65)
5	17	11	−0.09 (−0.66 to 0.36)	0.08 (−0.22 to 0.49)	−0.14 (−0.59 to 0.30)	0.17 (−0.41 to 0.63)
6	18	11	0.28 (−0.51 to 0.80)	0.08 (−0.27 to 0.51)	0.15 (−0.35 to 0.55)	0.09 (−0.14 to 0.60)
7	16	11	0.08 (−0.36 to 0.66)	−0.17 (−0.57 to 0.34)	−0.04 (−0.49 to 0.51)	0.19 (−0.25 to 0.57)
8	17	11	0.10 (−0.61 to 0.50)	−0.20 (−0.64 to 0.19)	−0.28 (−0.68 to 0.04)	0.23 (−0.23 to 0.54)
9	12	9	0.25 (−0.40 to 0.63)	−0.17 (−0.64 to 0.36)	0.12 (−0.37 to 0.51)	−0.20 (−0.49 to 0.29)
11	10	10	−0.01 (−0.88 to 0.52)	0.03 (−0.39 to 0.51)	−0.12 (−0.68 to 0.29)	0.18 (−0.17 to 0.73)
12	10	10	0.37 (−0.84 to 0.74)	0.21 (−0.18 to 0.65)	0.06 (−0.29 to 0.53)	0.00 (−0.44 to 0.55)
13	10	10	0.38 (−0.57 to 0.77)	0.01 (−0.33 to 0.39)	−0.01 (−0.51 to 0.57)	0.39 (−0.54 to 0.74)

a QOL: quality of life.

b Weeks from QOL: temporal correlations.

c Correlation (absolute value): small, *r*=0.1; medium, *r*=0.3; and large, *r*≥0.5.

### Correlation Between MH and Daily Smartphone GPS Data Over a 12-Week Period

Table 3 illustrates the temporal correlation between MH and weekly GPS location features. Correlations of −0.3 or higher were notable between time spent at home and MH at weeks 4 and 2 before the QOL assessment, as well as at week 9 after the QOL assessment, suggesting a potential link between increased time spent at home and lower MH. Correlations of ±0.3 or higher were significant between transition time and MH at weeks −4 and −2 before the QOL assessment, as well as at week 4 after the QOL assessment. Negative correlations at weeks −4 and −2 imply that increased transition time correlates with lower MH, while positive correlations at week 4 suggest a potential link between increased transition time and higher MH. Positive correlations of +0.3 or higher were significant between the number of significant locations and MH at 2 weeks before the QOL assessment and at week 13 after the QOL assessment, suggesting a potential link between an increased number of places visited and higher MH (Figure 2).

**Table 3.** Correlation between mental health and daily smartphone GPS features, including total distance, time spent at home, transition time, and number of significant locations, averaged by week and using 95% cluster-bootstrap CIs over a 12-week period.

From QOL^a^, weeks^b^	Observations, n	Participants, n	Total distance mean, *r^c^* (95% CI)	Time spent at home mean, *r* (95% CI)	Transition time mean, *r* (95% CI)	Number of significant locations mean, *r* (95% CI)
−6	11	10	−0.03 (−0.50 to 0.38)	0.47 (0.07 to 0.77)	0.44 (0.07 to 0.87)	−0.11 (−0.35 to 0.29)
−5	11	10	−0.18 (−0.67 to 0.1)	0.43 (0.13 to 0.78)	0.24 (−0.01 to 0.8)	0.11 (−0.21 to 0.6)
−4	12	10	0.09 (−0.39 to 0.34)	−0.03 (−0.35 to 0.42)	0.05 (−0.48 to 0.59)	−0.39 (−0.75 to 0.02)
−3	16	11	0 (−0.33 to 0.41)	0.02 (−0.23 to 0.39)	0.08 (−0.31 to 0.51)	0.04 (−0.38 to 0.49)
−2	16	10	−0.16 (−0.51 to 0.27)	0.07 (−0.3 to 0.28)	0.16 (−0.15 to 0.37)	−0.2 (−0.56 to 0.16)
−1	19	11	−0.1 (−0.49 to 0.28)	0.3 (−0.08 to 0.62)	0.3 (−0.05 to 0.66)	−0.21 (−0.41 to 0.02)
0	19	11	−0.13 (−0.45 to 0.16)	0.14 (−0.15 to 0.38)	0.08 (−0.32 to 0.45)	−0.18 (−0.55 to 0.18)
1	18	11	−0.28 (−0.6 to 0.04)	0.13 (−0.25 to 0.47)	−0.11 (−0.45 to 0.22)	0 (−0.42 to 0.42)
2	19	11	0.05 (−0.24 to 0.35)	0.19 (−0.13 to 0.48)	−0.02 (−0.47 to 0.44)	−0.09 (−0.46 to 0.35)
3	20	11	0 (−0.4 to 0.41)	0.05 (−0.29 to 0.4)	0.18 (−0.3 to 0.62)	−0.49 (−0.65 to‐0.09)
4	16	11	0.13 (−0.28 to 0.54)	0.24 (−0.09 to 0.51)	0.61 (0.26 to 0.84)	0.09 (−0.57 to 0.36)
5	17	11	0.15 (−0.3 to 0.65)	0.24 (−0.04 to 0.45)	0.28 (−0.09 to 0.52)	−0.11 (−0.62 to 0.25)
6	18	11	0.24 (−0.23 to 0.67)	0.22 (0.01 to 0.55)	0.27 (−0.01 to 0.53)	0.05 (−0.36 to 0.57)
7	16	11	0.19 (−0.29 to 0.68)	0.19 (−0.21 to 0.47)	0.31 (0.08 to 0.61)	−0.3 (−0.65 to 0.05)
8	17	11	0.15 (−0.51 to 0.62)	0.14 (−0.38 to 0.5)	0.13 (−0.41 to 0.44)	0.01 (−0.85 to 0.39)
9	12	9	0.33 (0 to 0.83)	−0.2 (−0.71 to 0.58)	0.19 (−0.36 to 0.69)	−0.43 (−0.77 to 0.07)
11	10	10	0.23 (−0.52 to 0.85)	0.39 (−0.23 to 0.82)	0.36 (−0.43 to 0.76)	−0.32 (−0.8 to 0.59)
12	10	10	0.34 (−0.3 to 0.77)	0.46 (−0.05 to 0.86)	0.54 (0.14 to 0.87)	−0.48 (−0.87 to 0.03)
13	10	10	0.33 (−0.15 to 0.94)	0.23 (−0.31 to 0.71)	0.2 (−0.31 to 0.7)	0.33 (−0.9 to 0.77)

a QOL: quality of life.

b Weeks from QOL: temporal correlations.

c Correlation (absolute value): small, *r*=0.1; medium, *r*=0.3; and large, *r*≥0.5.

## Discussion

### Principal Findings

The findings of this exploratory, secondary analysis demonstrate the potential association between smartphone-derived GPS-based movement features and QOL of patients with advanced cancer and their family caregivers. While not powered for statistical significance, correlations were observed between GPS movement variables and PH and MH during stretches of time before and after PROM assessment. These results warrant continued investigation of the potential of digital phenotyping approaches to passively assess patient and caregiver QOL in the context of cancer. Future analyses could investigate the interrelationship between the movement patterns and QOL of both patients and their caregivers, offering deeper insights into the dynamics between their experiences.

This study extends the existing body of research associating higher movement with higher QOL for patients with cancer and their family caregivers [45]. Notably, significant correlations were observed between PH and the total distance covered in GPS data collected 3 weeks before the QOL assessment. This indicates a potential connection between higher PH and longer distances traveled during this period. Additionally, our study findings align with previous research, indicating a correlation between PH and time spent at home [45,46]. Our findings suggest that poor PH is associated with increased time spent at home at 2, 3, and 4 weeks before the QOL assessment. This finding is supported by earlier research, which has proposed that an extended duration at home might be indicative of fatigue or impaired motor function [45]. However, our study findings diverge from prior research on the correlations between PH and the transition time [47], or the number of significant locations [48]. While our results suggest that decreased mobility is associated with higher PH, earlier research has proposed that increased mobility is an indicator of recovered PH. These findings imply that the metrics of transition time and number of visited places may not adequately capture how these indices relate to PH. Given the different contexts of the study design, it is not surprising that our results were inconsistent with the findings of a previous study. Future research may, therefore, explore the context of the relationship between PH and GPS measures in further detail, such as clinical populations or specific environmental contexts.

Our study did not reveal significant correlations between MH and the time spent at home or the number of significant locations overall. However, specific weeks within the 12-week period showed noteworthy correlations. For example, a statistically significant correlation emerged at 1, 5, and 6 weeks when GPS data were collected before the QOL assessment, indicating an association between increased time spent at home and MH. Similarly, at 3, 7, 9, 10, 11, 12, and 13 weeks, when GPS data were collected after the QOL assessment, a significant correlation emerged, suggesting a link between the number of significant locations and MH. These findings indicate temporal variability in the relationship between individuals’ daily activities, such as time spent at home and various locations, and their MH across distinct phases. While the precise significance of these periodic correlations remains uncertain, it underscores the necessity of accounting for temporal variations in understanding the connections between environmental exposure and MH outcomes. Further investigation is warranted to explore potential contributing factors to these temporal variations and to elucidate the nuanced dynamics of the interplay between daily routines and MH across different time frames.

Moreover, in our exploration of the relationship between MH and movement, we found that the total distance exhibited either no correlation or only a small correlation over time. Existing literature presents mixed findings; however, a significant portion indicates that traveling shorter distances is associated with worse outcomes in terms of emotional and cognitive well-being [49-51]. When exploring the association between MH and transition time, our results displayed mixed outcomes, aligning with previous studies where results on the correlation between MH and movement conflicted [39,52]. Notably, 1 study indicated that individuals with irregular movement patterns—described by irregularity features such as variations in circadian movement and routine index across days—tended to report fewer depressive symptoms and less loneliness compared to those with more regular patterns, hinting at a potential link between irregular movement and improved overall MH [53]. While our findings suggest that distance and movement behaviors may not be directly linked to specific MH aspects, they contribute to the broader understanding of general movement patterns, such as total distance and transition time.

### Limitations

This study has several limitations. First, the small sample size introduces uncertainty in correlation estimates and limits the generalizability of the findings. Future research with a larger sample size is needed to validate and extend these findings. Second, the participant sample was recruited from a specific group of patients with advanced cancer and family caregivers attending a particular oncology clinic, raising the potential for selection bias. Future research endeavors should strive for a more geographically and racially diverse sample. Third, the pilot study encountered issues with missing sensor data, leading to the exclusion of weeks with less than 3 days of data and days with less than 10 hours of data per day from the analysis. This limitation narrows the generalizability of our findings for those periods. The exact cause of the missing data is unclear; it could be attributed to several factors, such as technical issues with the app or the participants’ devices not being turned on, potentially due to reasons such as the device not being charged. Fourth, GPS data alone does not provide specific information about the type of activity performed outside the home, which limits the interpretation of the data and its correlation with quality-of-life questionnaires. Although we extracted several features from the GPS data to offer insights into participant behavior (eg, total distance traveled and time spent at home), the inability to classify activity types outside the home remains a limitation in the analysis. This limitation could impact the accuracy of the behavioral patterns examined in relation to QOL. Despite these acknowledged limitations, the study suggests the potential integration of digital phenotyping into health research, providing valuable insights into the challenges faced by individuals with complex health conditions and their caregivers in their daily lives.

### Conclusion

This study investigated the correlation between the QOL of patients with advanced cancer and family caregivers and smartphone GPS data. The results revealed complex relationships between smartphone GPS features and participants’ PH and MH outcomes. Specifically, total distance, time spent at home, transition time, and the number of significant locations showed various correlations with reported QOL. The study contributes to the field of digital phenotyping by shedding light on the intricate dynamic relationships between daily behaviors captured through smartphone GPS data and well-being among family caregivers and patients with advanced cancer. The findings emphasize the importance of considering personalized patterns and contextual factors when interpreting the impact of smartphone sensor data on health outcomes.

## Supplementary material

10.2196/59161 Multimedia Appendix 1 Participant-reported quality of life.

10.2196/59161 Multimedia Appendix 2 Daily smartphone GPS location features averaged by week.

## Abbreviations

IRB: institutional review board
MH: mental health
PH: physical health
PROM: patient-reported outcome measure
PROMIS: Patient-Reported Outcomes Measurement Information System
QOL: quality of life
REDCap: Research Electronic Data Capture
