# Supplementary material for: Exploring the Relationship Between Smartphone GPS Patterns and Quality of Life in Patients With Advanced Cancer and Their Family Caregivers: Longitudinal Study
Source: JMIR Form Res. 2025 Feb 7;9:e59161. doi: 10.2196/59161 (PMC11830490; doi:10.2196/59161)
Supplement: Multimedia Appendix 1 [file formative-v9-e59161-s001.docx]

|  | **Physical Health (T-Score)** | | | **Mental Health (T-Score)** | | |
| --- | --- | --- | --- | --- | --- | --- |
|  | **Caregivers**  **(n = 7)** | **Patients**  **(n = 4)** | **All**  **(N = 11)** | **Caregivers**  **(n = 7)** | **Patients**  **(n = 4)** | **All**  **(N = 11)** |
| **Baseline** |  |  |  |  |  |  |
| Mean (SD) | 45.43 (4.55) | 50.08 (9.37) | 47.12 (6.66) | 47.89 (7.72) | 55.5 (8.83) | 50.65 (8.60) |
| Median  (Range) | 44.8  (39.7, 54.1) | -  (39.7, 62.3) | 44.8  (39.7, 62.3) | 45.7  (38.9, 59.4) | -  (45.7, 67) | 50.5  (38.9, 59.4) |
| PH^a^ <42, n (%) | 1 (14.29) | 1 (25) | 2 (18.18) |  |  |  |
| MH^b^ <40, n (%) |  |  |  | 1 (14.29) | 0 | 1 (9.09) |
| **6-weeks** |  |  |  |  |  |  |
| Mean (SD) | 50.17 (10.33) | 47.05 (9.16) | 49.04 (9.57) | 51.13 (8.96) | 52.2 (6.02) | 51.52 (7.70) |
| Median  (Range) | 44.8  (39.7, 67.2) | -  (37.3, 58) | 44.8  (37.3, 67.2) | 50.5  (38.9, 67) | -  (43.4, 56.1) | 53.2  (38.9, 67) |
| PH < 42, n (%) | 1 (14.29) | 1 (25) | 2 (18.18) |  |  |  |
| MH <40, n (%) |  |  |  | 0 | 0 | 0 |
| **12-weeks** |  |  |  |  |  |  |
| Mean (SD) | 46.83 (6.07) | 46.3 (9.06) | 46.64 (6.84) | 52.21 (7.27) | 53.93 (8.13) | 52.84 (7.23) |
| Median  (Range) | 44.8  (39.7, 58) | -  (37.3, 54.1) | 44.8  (37.3, 58) | 53.2  (38.9, 59.4) | -  (43.4, 63) | 53.2  (38.9, 63) |
| PH < 42, n (%) | 1 (14.3) | 2 (50) | 3 (27.27) |  |  |  |
| MH <40, n (%) |  |  |  | 1 (14.3) | 0 | 1 (9.09) |

^a^PH = Physical Health.

^b^MH = Mental Health.

^c^Lower scores (MH < 40, PH < 42) indicate poor mental and physical health,^[1]^ and are associated with higher risk of future healthcare utilization.^[2,3]^

References

1. Saeb S, Lattie EG, Kording KP, Mohr DC. Mobile phone detection of semantic location and its relationship to depression and anxiety. JMIR Mhealth Uhealth. Aug 10, 2017;5(8):e112. [doi: 10.2196/mhealth.7297] [Medline: 28798010]

2. Wang R, Wang W, Aung MH, et al. Predicting symptom trajectories of schizophrenia using mobile sensing. GetMobile Mobile Comp Comm. Sep 5, 2018;22(2):32-37. [doi: 10.1145/3276145.3276157]

3. Odom JN, Lee K, Currie E, et al. Feasibility and acceptability of collecting smartphone data to predict adverse outcomes using digital phenotyping among family caregivers and patients with advanced cancer (Forthcoming). JCO Clin Cancer Informatics.
