# Supplementary material for: Exploring the Relationship Between Smartphone GPS Patterns and Quality of Life in Patients With Advanced Cancer and Their Family Caregivers: Longitudinal Study
Source: JMIR Form Res. 2025 Feb 7;9:e59161. doi: 10.2196/59161 (PMC11830490; doi:10.2196/59161)
Supplement: Multimedia Appendix 2 [file formative-v9-e59161-s002.docx]

| **Week** | **Participants** | **Number of data days** | **Number of data hours/day** | **Total Distance [km]^a^** | **Time Spent at Home**  **[min]^a^** | **Transition Time [min]^a^** | **Number of Significant Locations^a^** |
| --- | --- | --- | --- | --- | --- | --- | --- |
|  | **N** | **Mean**  **(SD)** | **Mean**  **(SD)** | **Mean**  **(SD)** | **Mean**  **(SD)** | **Mean**  **(SD)** | **Mean**  **(SD)** |
| 1 | 8 | 5.88  (1.55) | 17.82  (3.56) | 117.87  (52.08) | 561.13 (249.18) | 844.51 (118.43) | 4.26  (0.91) |
| 2 | 7 | 6.43  (1.13) | 19.31  (3.08) | 102.17  (66.46) | 569.42 (257.48) | 860.49  (116.70) | 4.24  (1.01) |
| 3 | 10 | 5.90  (1.2) | 19.04  (3.12) | 186.71  (153.15) | 551.51 (253.31) | 903.35 (109.39) | 4.23  (1.35) |
| 4 | 10 | 6.30  (0.82) | 18.57  (3.37) | 152.53  (94.26) | 562.49 (247.32) | 858.40 (146.59) | 5.08  (1.55) |
| 5 | 10 | 6.90  (0.32) | 19.19  (3.31) | 143.41  (141.38) | 573.25 (271.11) | 833.7  (156.29) | 4.55  (1.13) |
| 6 | 10 | 6.20  (1.32) | 18.77  (3.67) | 157.19  (103.96) | 573.24 (311.40) | 887.40 (145.17) | 4.56  (1.13) |
| 7 | 11 | 5.55  (1.86) | 19.08  (3.27) | 108.63  (71.25) | 437.18 (388.49) | 800.44 (225.31) | 4.52  (1.45) |
| 8 | 10 | 6.50  (0.71) | 18.73  (3.28) | 157.70  (117.31) | 481.57 (319.43) | 860.14 (111.51) | 4.63  (1.27) |
| 9 | 11 | 6.45  (1.21) | 18.54  (3.2) | 110.49  (64.59) | 522.58 (254.54) | 861.19 (122.19) | 4.36  (1.02) |
| 10 | 11 | 6.45  (0.93) | 18.79  (2.9) | 115.20  (89.91) | 553.13 (252.49) | 862.10 (132.40) | 4.64  (0.72) |
| 11 | 11 | 6.64  (0.5) | 18.70  (2.84) | 105.09  (81.27) | 489.49 (275.19) | 821.50 (133.41) | 4.41  (1.07) |
| 12 | 11 | 6.55  (1.51) | 18.10  (3.3) | 110.87  (85.50) | 520.10 (265.43) | 851.19 (137.23) | 4.47  (1.21) |

^a^Computed after extrapolation of available data to 24 hours.
